# Perceptions and Use of Teaching Strategies for Fundamental Movement Skills in Primary School Physical Education Programs

**DOI:** 10.3390/children9020226

**Published:** 2022-02-08

**Authors:** Danielle Salters, Sara M. Scharoun Benson

**Affiliations:** Department of Kinesiology, University of Windsor, 401 Sunset Ave, Windsor, ON N9B3P4, Canada; saltersd@uwindsor.ca

**Keywords:** spectrum of teaching styles, motor development, teachers, motor skills, motor learning

## Abstract

Fundamental motor/movement skills (FMS) describe the basic skills necessary to complete physical tasks, and are a key aspect of primary school physical education (PE) programs. Yet, specific teaching styles for FMS development have been relatively unexplored. Through a mixed-methods design, experiences and perceptions of different PE teachers (preservice, specialist, and generalist) were explored. The Spectrum of Teaching Styles (STS) survey was used to quantify self-reported use of teaching styles that may be used by PE teachers (*N* = 102). Semi-structured, qualitative interviews with a subset of participants (*N* = 11) were employed to explore how PE teachers perceive FMS development in PE classes. Combined, the findings highlight a preference for collaborative approaches to teaching and learning in PE, with a specific preference for explicit teaching strategies. Survey results demonstrated a preference for Style B (the practice style), which promotes teacher facilitation of activities and constructive feedback, with opportunities for students to practice skills and receive feedback. Teachers described how confidence with PE content influences the ability to provide lessons that target FMS development; this was reinforced by desires for additional professional development and training. Together, the findings provide a holistic view of teaching styles used in PE for FMS development, and outline a need to explore teaching approaches used by different PE teachers.

## 1. Introduction

Fundamental motor/movement skills (FMS) are the building blocks of more complex skills, impacting motor learning and the acquisition of more complex and dynamic movements to facilitate participation in physical activity (PA) at various levels [1]. FMS have been described as the foundational skills that must be learned in order to participate in more complex physical activities [2]. There are three categories of FMS: (1) locomotor skills involving movement of the body across a space (e.g., running), (2) object control/manipulation skills (e.g., catching or throwing a ball), and (3) stability and balancing skills (e.g., standing on one foot; [3]). Children who do not receive adequate motor skill instruction may experience developmental delays in the acquisition of gross motor skills [4].

Globally, there has been a declining trend in motor proficiency, with less than 50% mastery in locomotor and object-control skills, and only 11% displaying advanced proficiency among 12- to 13-year-old children [5]. More recently, a systematic review of the literature (60 articles, examining the FMS proficiency of over 21,000 children, aged 3–10 years, in 25 countries) demonstrated average locomotor proficiency among 57–64% of children, average object-control proficiency among 51–69% of children, and an overall average FMS competency among 34–49% of children [6]. The development of FMS is essential to participation in PA across the lifespan, both in traditional and non-traditional contexts (for example, cycling and aquatic activities have generally not been considered traditional in the FMS literature [7]). A Canadian study [8] found positive correlations between FMS proficiency and participation in recreational PA across a 20-year period. Individuals with higher FMS proficiency spent less time in sedentary pursuits [8]. These studies demonstrate a need to investigate how skills are being taught, as well as how FMS development and PA is promoted.

Traditional FMS are a pedagogical focus in Physical Education (PE) programs, incorporating FMS development into curriculum expectations. For example, the Ontario curriculum incorporates FMS with Movement Competence. This strand focusses on transferrable skills (i.e., an overhand throw can be used in ball games, such as baseball, but the motion is also useful for an overhand serve in tennis) through understanding of the three movement phases (preparation, execution, and follow-through) and how to apply these movements to other activities [9]. PE teachers play a critical role in the development of physical literacy (the confidence and competence to apply a variety of physical skills in a number of different physical contexts [10]) during primary school through appropriate activities that promote motor competence and the ability to participate in and accomplish increasingly complex tasks. Teachers are poised to scaffold lessons in such a way that FMS are acquired before advancing to these complex tasks, and ensure that students achieve success before moving to the next stage of skill complexity [9]; for example, students must first learn how to jump and land properly before they can learn how to do a long-jump in a track and field event context.

PE teachers are important agents in facilitating skill development in children. Mosston and Ashworth’s [11] Spectrum of Teaching Styles (STS) describes 11 teaching styles used in PE lessons, ranging from Command Style (teacher-centered), to Self-Teaching Style (student-centered). Explicit learning styles, housed in the reproductive cluster of the STS, describe styles where the teachers make the majority of the decisions about learning activities, including content and task progression (teacher-centered). Conversely, implicit styles, characteristic of the productive cluster, describe styles where students have the opportunity to make decisions about their own learning activities (student-centered; [12]). Generally, teacher-centered styles have been positively associated with increased motor learning and skill development, while student-centered styles have not [13]. Despite the relationship to skill development, these implicit learning styles have been found to be beneficial for individuals with underdeveloped cognitive resources, or those with difficulties with working memory [14]. Implicit teaching styles have generally used games that facilitate skill development with a manipulated practice environment to reduce student errors during learning [15].

Despite the importance of FMS in overall physical development, consideration of specific teaching styles in PE and FMS development has been largely unexplored. The purpose of this study was to explore teacher perceptions about teaching styles used in primary school PE to develop FMS, and determine whether explicit or implicit learning activities are perceived more favourably in order to facilitate the development of FMS in primary school-aged children.

Unique to the current research, this study examined similarities and differences among preservice teachers, specialist primary school PE teachers, and general primary school teachers. Each of these groups has different knowledge and experiences with PE and the facilitation of skill development in primary school-aged children. In Ontario, PE has been delivered predominantly by generalist teachers, despite the presence of specialists who have completed a major or minor in PE (average of 3–5 years) before completing a Bachelor of Education. School leadership has expressed doubts in having generalists teach PE [16], with agreement among generalists towards a lack of confidence in their abilities, related to a lack of skill and knowledge about planning and delivery [16,17,18].

Specialist PE teachers are advantageously placed to ensure that PE lessons receive quality planning, teaching, and learning, while ensuring continuity and progression as the student develops and grows. Despite being aware of the benefit of specialists, only a small number of schools employ specialist PE teachers [16]. Differentiating between teacher groups is thus essential to obtaining a comprehensive view of teaching and learning strategies that are used most often in primary school PE lessons. Taken together, examination of the three groups of teachers’ self-reported use of the STS and preferences for implicit or explicit learning activities provides a holistic view of teaching FMS in primary school PE, and promotes an understanding of the differences between the preservice, specialist primary school PE, and general primary school teachers in a PE context.

The current research examined primary teachers’ (preservice, specialist, generalist) self-reported use of the STS in PE and preferences for implicit or explicit learning activities. A mixed-methods approach was employed. The quantitative portion examined participants’ self-reported teacher styles, guided by the following questions: (1) What teaching styles within the STS do primary PE teachers self-report as using? (2) How do different experience levels of PE teachers (preservice, specialist, and generalist) differentiate on self-reported use of STS? It was hypothesized that teachers would report using teaching styles within the reproductive cluster (teacher-directed/explicit) of the STS more often than those within the productive cluster (student-directed/implicit) to guide PE lessons [12,13,19,20,21]. The qualitative section focused on teacher perspectives of FMS and reflections on how FMS is taught within PE. This section was guided by the following questions: (3) What are the perspectives of primary school teachers on FMS development? (4) How do PE teachers in primary school perceive FMS development as related to explicit and implicit teaching styles? Due to the explorative nature of this qualitative section, no hypotheses were formulated [22].

## 2. Materials and Methods

To account for the broad and dynamic nature of teaching, a mixed-methods approach was employed. Through the use of a triangulation mixed-methods design [23], qualitative interview data augmented a quantitative survey in order to explore how self-reported teaching styles relate to PE teachers’ perceptions of FMS development in primary school-aged children. A holistic approach was used to garner deeper understanding of the perceived teaching strategies for FMS through unique teacher insights and perspectives collected through the survey and interview independently, as well as collectively [24,25]. This approach was considered crucial to this project in order to explore perspectives of preservice and in-service teachers through two distinct ways of thinking about the phenomena (teaching strategies for skill development in primary school PE [23]). Quantitative data were gathered to assess self-reported teaching styles in PE; survey questions were based on the STS framework, and the questionnaire developed by Kulinna and Cothran [21]. Semi-structured qualitative interviews were conducted with a subset of participants (all survey participants were invited to participate; interviews were conducted with volunteers) to further understand participant knowledge and experiences [26] and to explore teacher perspectives of FMS and the impact of explicit and implicit learning. Data were collected from three groups of teachers with distinct experiences and training in delivering PE lessons: (1) preservice teachers enrolled in a teacher training program, (2) primary specialist PE teachers who primarily teach PE, and (3) primary general teachers who teach in a general classroom (all subjects), but have taught or currently teach 1–2 PE sessions in the week. Ethics clearance was obtained from the University of Windsor Research Ethics Board (REB# 20-124).

### 2.1. Method 1—Survey

#### 2.1.1. Participants

Of the 137 collected responses, 35 were removed due to incomplete survey responses (see the Results section for more details) or a failure to meet the eligibility criterion (see procedures for more details). With these considerations, data from 102 participants were included (Table 1).

#### 2.1.2. Procedures

Pre-service teachers were recruited via an email sent from the Faculty of Education’s administration. Specialist and generalist teachers were recruited using purposive and snowball sampling through social media groups. An invitation link directed potential participants to the “Physical Education Teachers’ Perceptions of Teaching Styles” [21] instrument, hosted on Qualtrics; the landing page was the information letter and consent form. Multiple-choice pre-screening questions determined participant eligibility based on requisite knowledge: (1) What is PE? (2) True or False: The Ontario Curriculum includes Health and PE (HPE), (3) Which of these is not a strand in the Ontario HPE Curriculum? (4) True or False: Safety skills are included in the Active Living strand of the curriculum, and (5) True or False: Living Skills are an additional strand in the curriculum that contributes to student success in other strands. Participants were informed that incorrect answers would result in disqualification from the study due to inadequate prior knowledge of teaching practices in PE. Only eligible participants were invited to complete basic demographic questions and the survey instrument to assess teacher’s self-reported teaching styles according to the STS framework [13,21,27].

The 11 teaching styles proposed in the STS were individually assessed in a mixed order (G—Convergent Discovery; D—Self-Check,; J—Learner Initiated; A—Command; F—Guided Discovery; C—Reciprocal; I—Learner Designed; E—Inclusion; H—Divergent Discovery; B—Practice; K—Self-Teaching), with a brief scenario for each, followed immediately by the following four statements: (a) I have used this way to teach PE, (b) I think this way of teaching would make class fun for my students, (c) I think this way of teaching would help students learn skills and concepts, (d) I think this way of teaching would motivate students to learn. Participants responded using a 5-point Likert scale (1 = strongly disagree, 5 = strongly agree). Favourable perspectives were characterized by higher average scores of the four questions. This instrument is highly reliable, with Cronbach’s alpha coefficients between 0.86 and 0.91, validity scores measured with eigenvalues for the 11 teaching styles between 7.11 and 1.05, and structure coefficients between 0.78 and 0.90 [21].

#### 2.1.3. Data Analysis

Data were analyzed using SPSS (IBM^®^) statistical software (version 25). Independent measures were participant age, gender, setting, and teacher type. Dependent measures were teaching styles within the STS. Likert data were averaged to obtain a single score per style, and subsequently, to obtain the average for teaching styles within the reproductive and productive clusters, respectively (e.g., [21]). Two reliability tests were performed for each cluster of the STS: for the reproductive cluster, a Cronbach’s alpha score of 0.649 demonstrated moderate reliability, while a score of 0.521 also demonstrated moderate reliability for the productive cluster [28,29,30,31]. Two multiple regression tests were run to predict participant preferences of use of the reproductive or productive cluster of teaching styles based on participant age, gender, setting, and teacher type (e.g., [27]). However, similar to Kulinna and Cothran [21], the four items for teach teaching style were used to gain general impressions of participants towards each of the teaching styles. With limited participants aged 36–45 (*n* = 5), 46–55 (*n* = 3), and 56–65 (*n* = 1), participants were grouped 18–25 (*n* = 42) and over 26 years (*n* = 60). With a limited number of specialist PE teachers (*n* = 10), analyses were performed twice: (1) comparing the three groups, recognizing the violation of statistical assumptions, and (2) comparing preservice teachers to in-service teachers (i.e., generalists and specialists merged into one variable).

### 2.2. Method 2—Interviews

#### 2.2.1. Participants

This research included 11 participants (6 females, 5 males, ages 24 to 50, *M* = 28.91 ± 7.59), including preservice teachers (*n* = 2), generalist primary teachers (*n* = 7), and specialist PE teachers (*n* = 2). Experience teaching (in any capacity) ranged from 0 (preservice) to 21 years (*M* = 4, *SD* = 5.92). Of note, the audio recording for one participant was corrupted, therefore, their data relied on interviewer notes.

#### 2.2.2. Procedures

Upon completion of the survey, participants were asked to participate in follow-up interviews, which resulted in 11 volunteers. Interviews were conducted over Zoom or Microsoft Teams with the first author. Before interviews, participants were assured of confidentiality, informed that all answers were based on their own experiences and perspectives, and that they could skip questions or stop the interview at any time without consequence. With permission, interviews were audio-recorded. Recordings were downloaded and deleted from the application server upon completion. Interviews ranged between 25 and 50 min. To thank them for participating, a $10 gift card was provided.

Interviews sought to examine the lived experiences of PE teachers as they relate to explicit and implicit teaching strategies for FMS in children, and thus were guided by exploratory analysis and phenomenology. Exploratory analysis promotes the description or understanding of a specific phenomenon through the use of participants’ interpretations and perspectives [32,33]. Exploratory analysis uses inductive reasoning to come to conclusions based on collected data and the formation of new ideas and concepts, and was used to relate teaching styles within the STS to the teacher’s perceived use of explicit or implicit teaching strategies [34]. Phenomenology focusses on shared lived experiences of a group of individuals [35], and assumes that subjective experiences can be interpreted as certainties for the individual [36,37]. Descriptive phenomenology was used to promote discussion about teaching strategies and FMS development through personal perspectives and experiences of the interview participants [36,37]. Through a phenomenological approach, participants were encouraged to reflect on their attitudes and interpretations of the phenomena [38]. Interviews were semi-structured in nature to allow for follow-up questions [37]; questions were centered around perceptions of FMS in primary school-aged youth, preferred teaching styles, and whether explicit or implicit learning activities have the strongest perceived influence on the development of FMS.

Interviews were transcribed verbatim on Microsoft Word, where identifying information and language errors were removed to facilitate readability. Completed transcriptions were sent to participants for member-checking, to ensure accurate representations of the experiences and opinions of participants [39]. For the corrupted interview, the general observations from interviewer notes were sent to the participant for member-checking. Finalized transcriptions were thematically analyzed using NVivo (2020) to identify key commonalities across teacher styles and experience levels. Three guiding questions were used during thematic analysis [37]: (1) What are the participants expressing during the interview? (i.e., What are interview data telling us about the strategies preferred for teaching specific FMS? Are participants offering similar perspectives and opinions?); (2) What is it that I want to know? (i.e., Do participants express a preference for specific teaching strategies for FMS development? Are there differences in perceptions based on the type of PE teacher?); and (3) What are the implications of the participant’s expressions and interpretations? (i.e., What do the interview data tell us about teaching strategies and FMS development?). Supplementary analysis compared teacher perspectives among the different types of PE teachers, and to ensure that data collection took place until saturation of the themes was met [40]. Saturation was determined by the inability to identify new and emerging themes from the coded transcriptions and to avoid informational redundancy in the data [40,41]. Data saturation was assessed both during data collection (through reflexive journal entries) and following data analysis (no new emerging themes throughout analysis of transcribed interviews; [41]). Upon completion of data collection and coding, the second author went through coded data to check the interviews to ensure that emerging themes were true representations of the data.

## 3. Results

### 3.1. Survey Results

There were multiple missing data points due to participants ending the questionnaire early or skipping over specific questions (participants were excluded if they missed over 40% of the questions—four or more of the last teaching styles were unanswered; *N* = 30) or failure to meet eligibility criteria (*N* = 5). Little’s Missing Completely at Random (MCAR) test was used to test the hypothesis that missing item responses (22.5%) were missing completely at random (χ^2^(472) = 513.64, *p* = 0.090). Expectation-maximization was used to impute missing data (e.g., [42,43,44,45]).

#### 3.1.1. Reproductive Cluster with Pre-Service, Generalist, and Specialist Teachers

Multiple regression for the reproductive cluster were performed first with specialists included (recognizing that this population does not have appropriate numbers). There was independence of residuals, as assessed by a Durbin–Watson statistic of 2.051, and no evidence of multicollinearity, as assessed by tolerance values greater than 1.0. There were no studentized deleted residuals greater than ± 3 standard deviations, no leverage values greater than 0.044 (LeverageM = 0.011), and no Cook’s Distance above 0.085 (CooksM = 0.011). Assumptions for normality were met, as visually assessed by the Q-Q plot, and Shapiro–Wilks scores (*p* > 0.05). Age, gender, and teaching setting were classified as excluded variables. With teacher type as the only included predictor, the multiple regression model predicted statistical significance for perspectives towards use of the reproductive cluster of teaching styles: F(1, 94) = 12.617, *p* = 0.001, adjusted R^2^ = 0.109. Teachers who identified as being generalists to specialists were more likely to perceive more use of teaching styles in the reproductive cluster; B = 0.238. Regression coefficients and standard errors are presented in Table 2.

A 3 (between subjects: preservice vs. generalist vs. specialist) × 5 (within subjects: styles A through E for the reproductive cluster) multivariate analysis of variance (MANOVA) was run to assess the preferences for the use of specific styles. Homogeneity of variance assumptions were met (*p* > 0.05). Tests of between-subject effects for the five teaching styles were not significant: F(10, 192) = 1.423, *p* = 0.173; Wilk’s Λ = 0.865, partial η^2^ = 0.070. Examination of the means and standard deviations for each of the teaching styles indicated that specialists tended to have slightly higher scores towards these styles than generalists, who in turn had slightly higher scores than preservice teachers (Table 3).

#### 3.1.2. Reproductive Cluster with Pre-Service and In-Service Teachers

Multiple regression for the reproductive cluster within the STS was performed with generalists and specialists merged (i.e., in-service teacher). There was independence of residuals, as assessed by a Durbin–Watson statistic of 1.757, and no evidence of multicollinearity, as assessed by tolerance values greater than 1.0. There were no studentized deleted residuals greater than ± 3 standard deviations, no leverage values greater than 0.018 (LeverageM = 0.010), and no Cook’s Distance above 0.058 (CooksM = 0.010). Assumptions for normality were met, as visually assessed by the Q-Q plot, and Shapiro–Wilks scores (*p* > 0.05). Age, gender, and teaching setting were classified as excluded variables. With teacher type as the only included predictor, the multiple regression model predicted statistical significance for perspectives towards use of the reproductive cluster of teaching styles: F(1, 94) = 6.800, *p* = 0.011, adjusted R^2^ = 0.058. Teachers who identified as in-service were more likely to perceive more use of teaching styles in the reproductive cluster; B = 0.232 (Table 4).

A 2 (between subjects: preservice vs. in-service) × 5 (within subjects: Styles A through E for the reproductive cluster) MANOVA was run to assess the preferences for the use of specific styles. Homogeneity of variance assumptions were met (*p* > 0.05). Style C was excluded from further analysis, as the assumption for homogeneity was not met. Tests of between-subject effects for the five teaching styles were not significant: F(4, 97) = 1.898, *p* = 0.117; Wilk’s Λ = 0.927, partial η^2^ = 0.073. Examination of the means and standard deviations for each of the teaching styles indicated that in-service PE teachers tended to have slightly higher scores towards these styles than preservice teachers (Table 5).

#### 3.1.3. Productive Cluster Did Not Demonstrate Significant Results

Multiple regressions were run for the productive cluster both with and without specialists. None of the independent variables were entered into the equation in either instance.

### 3.2. Interview Results

Thematic analysis demonstrated core consistencies in four areas: (1) Interpretations and assessment of physical literacy (PL) were concurrent with the Ontario Curriculum; (2) Teaching approaches and strategies emphasized a collaborative approach to teaching in PE; (3) Understanding of FMS was influenced by preservice training and familiarity with PE; (4) FMS are important to development, with evaluations focussing on skill progression. One minor supplementary theme emerged, independent of the four major themes (i.e., not connected to the research question or targeted within the interview guide, but still important to highlight); specifically, the desire for additional professional development (PD) and training in PE.

Perspectives and experiences working in a PE environment demonstrated similarities and differences between specialists, generalists, and pre-service teachers. Central to all discussions was the importance of PE. While there were inconsistencies with participant impressions of how PE was situated within the school, all participants agreed that PE was essential for the healthy development of the child. The following describes major themes that emerged, highlights subthemes, and offers support with representative quotations.

#### 3.2.1. Interpretations and Assessment of PL Were Concurrent with the Ontario Curriculum

Participants’ interpretations of PL were largely consistent with the Ontario HPE curriculum (i.e., “Individuals who are physically literate move with competence and confidence in a wide variety of physical activities in multiple environments that benefit the healthy development of the whole person” [9]. One participant described PL as: “With the kids who are good, they are able to transfer the movement and the instructions really fast, where you see other children it’s a conundrum for them and they have no idea what you want them to do” (P6). While most participants expressed that they had not been explicitly introduced to the concept of PL, explanations that addressed competence and confidence were provided:

“When you look at it, a lot of students aren’t comfortable throwing a ball, aren’t comfortable catching a Frisbee or doing different types of movement. I think that needs to be pushed a little bit more, because if students are comfortable and confident doing it that’s one thing, but then you always have that group of kids that don’t want to play sports, they aren’t really comfortable throwing a ball, kicking a ball. Maybe if we’re able to foster that confidence in them, they’ll be more willing to participate in PA”.

(P1)

Notably, participants acknowledged that ensuring skills are appropriate for the students would be key in promoting the development of PL in various age groups. There was a consensus that understanding the students and their various ability levels would facilitate the development of PL and promote further skill development during PE lessons: “For those students that feel like you have experience in this, you can go to level 2, for those students who’ve shown me that they’re good and confident you can go to level 3” (P4).

To determine the PL of the students, participants outlined a need to observe skill execution in different movement contexts. “[If] they can jump in basketball, but they can also jump in long jump, and they can also jump in soccer, and they can use the jumping skill competently in wide ranges or the whole entire physical movement, as well as being confident in it” (P10). Participants acknowledged that observations do not have to occur within formal PE lessons, but could be incorporated into a variety of daily activities. While this would be a beneficial approach for generalists, who spend the majority of the day with one group, this may prove challenging for specialists, whose only chance to observe the skills and development may be during PE.

#### 3.2.2. Teaching Approaches and Strategies Emphasized a Collaborative Approach to Teaching in PE

Participants expressed a range of teaching strategies that they may use. Student-centered approaches were viewed as those that incorporated higher levels of student activity, and practice of the skills being taught. For example, “I think with PE it’s a lot of go and do it, go and try it, give it a shot, and I will just throw a bone out there to you and say why don’t you try this, why don’t you take a look at this and see if that leads them to a new learning intention” (P3). Teacher-centered approaches were viewed as more prescriptive in nature, where the instructor clearly outlines the activities and expectations. For example: “You would have set and clear boundaries and expectations for students entering the space that you’re accessing. As well students will have clear direction of where they’re supposed to be, and what they will be doing” (P7).

Preference for a collaborative approach emerged consistently. The teacher served as the primary authority in the classroom, yet students were still able to make themselves heard in order to contribute to their learning; “Taking their opinions or advice into consideration, but ultimately, it would come down to my decision, so I would like to say a 70-30 teacher- vs. student-based approach” (P10). Interestingly, specialists (*n* = 2), and the majority of generalist teachers (*n* = 5) were open about preferences for a teacher-centered approach when compared to preservice teachers: “When I’m teaching a PE lesson, it is pretty much centered around me, what I want them to do, me modelling quite a lot of times what they are supposed to be doing, and me watching over that they’re doing what they’re supposed to be doing” (P8).

Participants outlined that polarized approaches are not conducive to learning in PE. Strongly teacher-centered approaches reduce the amount of time that students can spend actually being physically active: “It just takes up so much time of you just talking and the kids are just sitting there staring at you rather than actually practicing it themselves” (P3). Similarly, strongly student-centered approaches presented challenges related to trust and behaviour: “I would need a really good group of students that I know I could trust to be able to do things like that, and if it wasn’t the case then I would be doing a whole lot more teacher led activities” (P4). Collaborative efforts were situational. The participants’ willingness to allow for more student-centered activities was dependent upon the level of comfort and trust that they had with their students: “Would they be able to respect class rules without going out of hand. I think it really depends on the student that I teach rather than what I am teaching” (P2). Participants further indicated that they prefer to begin with a teacher-centered approach before allowing for student involvement and contribution: “When I’ve started it off, then I’m all for delegating responsibility to students that show an interest or have an ability as long as I have confidence that they will do it in a positive reinforcing way with the other kids. But, I want to start it out” (P6). In this way, the instructor could ensure students are aware of the lesson and contributes to learning.

Ensuring the students feel as though they can approach the instructor is key to promoting participation and engagement: “Having them know that if there is something they want to do, feel free to bring it up to me and we’ll see if we can incorporate it next time” (P1). While the main goals of the PE were to promote activity and skill development, student safety was always at the forefront of the lesson, regardless of preferred teaching strategies: “We get the basics, we get the safety parts, but then we start off with elements of the game. So, we get the ball rolling or the feet moving, or the heads tumbling as fast as possible” (P6).

#### 3.2.3. Understanding of FMS Was Influenced by Preservice Training and Familiarity with PE

When asked to outline their interpretations of FMS as a concept, explanations of FMS were varied and inconsistent with the FMS definition provided (i.e., FMS are the building blocks for complex movements which allow children to apply basic motor skills related to manipulation/object control skills, locomotor skills, and/or balance and stability in order to participate in a variety of physical activities; [1]). Specialists, both in-service (*n* = 2) and preservice (*n* = 1), had perceptions of FMS that were related to the specific skill categories (locomotion, manipulation/object control, and balance and stability), in order “to give them the building blocks to develop more complicated skills later” (P5). Generalists (in-service and preservice) tended to have a more functional interpretation: “Any type of movement that you would nearly require in everyday life” (P3). Despite differences, participants agreed that FMS were beneficial to activities for daily living, and were not limited to participation in PA pursuits.

#### 3.2.4. FMS Are Important to Development, with Evaluations Focussing on Skill Progression

FMS were seen as critical to growth and development during childhood. Discussions outlined that FMS are useful outside of PA and sport contexts. One participant stated, “The earlier that you learn them, and the more diversely that you can move your body, the younger that you are, I find that to be beneficial for any student” (P4). Similarly:

“I think a lot of people don’t realize how much they’re going to need the basic movement skills later in life. I think when people think of PE or think of PA, they’re pretty much stuck in a box that it has to be sport related, which it really isn’t. We need to be able to walk, to skip, to throw things, to catch things, because you use it every single day”.

(P1)

Participants highlighted that future intentions towards participation in PA and sport may be influenced by the development of FMS during childhood. “Awareness of their spaces, how their body moves and how they interact with things and other people. Determining how much they dedicate their life to PA and movement” (P7).

Extending beyond initial discussions of teaching methods, participants outlined that FMS instruction would begin with direct teaching styles (teacher-centered), as indirect styles (student-centered) would be useful for the students to hone their skill execution. Games were described as a useful strategy for practicing skills: “Make them build in complexity as they get older, depending on the group and what they can handle, and just have a lot of variety and make sure you include little bit of everything” (P5). Similarly, a participant described that breaking activities down into specific steps could be useful in facilitating FMS development:

“If they don’t get it, then we might have to give them some resources, such as have the steps written down in a checklist and see if they’re doing it, and break it down where they’re doing each individual step one at a time and putting it all together, and not all together at once, if there’s 5 steps and you just do 1,2,3,4 and 5 individually, and then 1 and 2, and then finally put it all together, so really breaking it down for that child”.

(P3)

Participants indicated that age-related benchmarks and standards are important for assessing where students should be: “I would be concerned if a child in grade 6 could not dribble a ball. I would be looking into that. … But there’s certain things you expect at certain ages that most kids can do” (P5). However, individual differences were also acknowledged:

“Even if you want to evaluate them generally, the children have different particular needs, and also, they have very different bodies, so children who may be longer and taller would have longer limbs so they would probably be much faster when they run compared to children who may be smaller with shorter legs”.

(P2)

Issues surrounding the use of benchmarks and standards were also addressed: “In grades 4–6 when kids start to hit puberty earlier than others, it would be unfair to grade them on certain standpoints for certain ages, and then just because this grade 7 can do this, they’re better than another” (P4). Participants generally agreed benchmarks and standards were useful to inform their teaching and what to look for at different stages of development, but that evaluations should be centered around skill progression: “If they’ve shown improvement, that means they’ve actually worked on the skill, then that’s what I count as a success” (P5). One participant further described that the teacher’s role is to ensure there are opportunities for students of differing ability levels: “It’s my job as a teacher to see where they’ve progressed… making sure I’m going back and taking a look at the concepts that [they] missed, and how I can get [them] to where [they] need to be now, and evaluating on the progress, not necessarily on the skill itself” (P10).

Confidence and competence with use of FMS in different contexts was highlighted as an additional component of evaluation, further demonstrating the connection between FMS and PL. For example: “I don’t think it needs to be evaluated more on the ability, because you’re going to have such a big range, but if they have the confidence to do it, and they want to incorporate those movement skills, or show how they can do those movement skills” (P1). Due to the individual nature of assessment, it is important for teachers to provide a variety of opportunities for all students to demonstrate success, beyond meeting age-related benchmarks.

#### 3.2.5. Desire for Additional PD and Training in PE

Participants highlighted that, despite the perceived importance of PE, there was a lack of PD or teacher training: “If it wasn’t part of my teachable, I was never taught movement or anything in teacher’s college” (P1). Regardless of training or subject specialty, all participants outlined benefits of additional PD in PE.

“Not even just for specialists, but for generalists, we always focus on math and language, and I think a lot of generalist teachers are maybe not sure about what they’re doing in PE, and I’m not sure that we have a consistent quality of education amongst all of the classes and schools in Ontario. Even though we’re all following the same curriculum, the way it’s interpreted and delivered might be very different”.

(P5)

Similarly: “I know there’s been more push for mental health, and there’s always a push for literacy and math, but why not do that for all the other subjects? They’re just as important and we all have something to contribute” (P7). When instructors do not receive enough training or PD there is a sense of learning on the job, and the necessity to seek out opportunities for learning and/or observation of best practices within the subject: “It’s always math, phonics, literacy or something else, but never for PE if you’re a general teacher, unless you request it yourself, but it’s often overlooked” (P8). Training and continuous learning were key concerns; participants outlined that the resources for PE (training programs, PD, and in-school mentors) are crucial to ensuring that instructors feel confident in their abilities to deliver effective lessons.

## 4. Discussion

The present research explored teacher perceptions of explicit (teacher-centered) or implicit (student-centered) teaching strategies to promote FMS development in primary school PE. Unique to this study, participants were grouped into three distinct categories: preservice, specialist, and generalist primary school teachers. Consistent with previous research, specialists were found to be more confident in their abilities to plan and deliver PE lessons, spending more time developing skills, providing various activities, and using different pedagogical approaches [46].

Through a mixed-methods design, four questions were used to guide the research. Through the quantitative-survey portion, the following questions were used: (1) What teaching styles within the STS do primary PE teachers self-report using? (2) How do different experience levels of PE teachers (preservice, primary PE specialist, and general primary school teachers) differentiate on self-reported use of STS? It was hypothesized that participants would self-report using teaching styles within the reproductive cluster of the STS (e.g., [12,13,19,20,21]). The qualitative interview portion was guided by the following questions: (3) What are the perspectives of primary school teachers on FMS development? (4) How do PE teachers in primary school perceive FMS development as related to explicit and implicit teaching styles? Due to the qualitative nature of the interviews, participants were free to express their perspectives, opinions, and experiences, and hypotheses were deemed inappropriate [22].

Together, survey and interview data provide a holistic view of perceptions for teaching and learning during primary school PE programs. Findings offer important insights regarding self-reported use of the STS and the perceived teaching strategies to facilitate FMS development.

The range of teaching styles within the STS provides teachers with a variety of approaches to teach skills in PE. It is important to note that the STS does not imply that any one style is better than another, or should be used in every instance. Rather, the goal of the STS is to provide teachers with a repertoire of tools that they can use to accommodate student needs, learning objectives, and lesson goals [11]. Analysis of STS data revealed teacher type was the only significant predictor of the reproductive cluster of teaching styles, with specialist PE and generalist teachers being more likely to prefer the use of these styles. Comparison between teacher groups was unique to this research, demonstrating need to further examine use of the STS among different teacher groups. Previous studies have found that preservice teachers demonstrate a preference for teaching styles within the reproductive cluster [13,21]. The use of the reproductive cluster of the STS has been found to be the most prevalent in PE settings [13,20,21]. Both male and female teachers have been found to prefer teacher-centered teaching styles, characteristic of the reproductive cluster in the STS [19,47], yet findings based on gender have been inconsistent (e.g., [13,21]). While gender was not a significant predictor in the current research, the finding that reproductive teaching styles were preferred by all genders remains consistent [12,13,21,47].

Further analysis of teaching styles within the reproductive cluster revealed the strongest preference for the practice style (Style B) within the STS; this finding is consistent with previous literature on preferences within the STS, indicating that the practice style is generally the most preferred style among in-service and preservice teachers (e.g., [13,20]. Within the practice style, the role of the teacher is to provide activities and learning outcomes, while decisions are shifted towards the student (e.g., when to initiate practice or interact with the task). The teacher observes students and provides individualized feedback during practice conditions [11]. Practice style has consistently produced the best outcomes for skill development [12], and has been highlighted as one of the most preferred teaching styles within PE [13,19,21]. Collectively, the quantitative and qualitative data support this finding, highlighting a collaborative approach to teaching and learning as the preferred strategy for PE teachers.

A collaborative approach to teaching and learning in PE was described as the teacher acting as a facilitator for the lessons, providing appropriate activities that promote skill development, and student participation. Within the PE setting, there are four major factors that may contribute to a collaborative learning environment: (1) teacher training (where teacher subject knowledge impacts the effectiveness of skill interventions), (2) class and behaviour management (to promote a positive environment), (3) learning interventions (feedback and/or observation to help students further develop skills), and (4) interventions for students with disabilities [48]. Teacher confidence to provide these activities is a major contributor; this is especially apparent among preservice and generalists who may lack training or knowledge to deliver effective PE lessons [17,49,50].

Participants highlighted that additional PD opportunities would be beneficial for increasing teacher confidence in their abilities to deliver effective PE lessons. Additionally, participants expressed a need to provide a positive learning environment that focusses on skill progression for all students. A comprehensive assessment requires both process- and product-based measures [51]. Participants generally preferred evaluations that focussed on skill progression (i.e., process-based) rather than meeting performance-related benchmarks (i.e., product-based). Process-oriented assessments allow the teacher to observe the various movement skills being assessed and identify aspects of the movements that should be targeted for further development or monitoring [52], compared to benchmarks or product-oriented assessments which look at the outcome of the movement only [2]. Challenges with process-oriented assessments, however, relate to the ability of the teacher to visually attend to all aspects of the movement and identify the aspects that require further monitoring [52]. When teachers favour an explicit/teacher-centered approach to skill teaching, they make most of the decisions, which leads to higher levels of skill proficiency, and successful skill execution [12,15]. Conversely, when there are opportunities for implicit/student-centered approaches, students have more opportunities to be involved in the decision-making process; this can be seen in Teaching Games for Understanding approaches, where skills are reduced or substituted, and modified games introduce students to the game principles [12]. Manso–Lorenzo et al. [53] found that explicit and implicit teaching strategies are not sufficient when used independently. Participants similarly outlined that a polarized approach to teaching PE would not be effective, and that a combination of both teacher- and student-centered approaches would be the most beneficial.

It is important to note that, independent of teacher type, other factors (age, gender, or teaching location) did not predict use of teaching styles. Qualitative findings support the notion that teaching practices are influenced by subject knowledge, experience, and training. PE requires unique subject and pedagogical knowledge in to confidently and appropriately plan lessons that facilitate FMS development (e.g., [54]). In Ontario, most primary schools have adopted a teaching model that uses generalists for PE more often than specialists [46]. Specialists have the requisite knowledge to provide effective PE lessons, compared to generalists who may have insufficient training [46]. Confidence for teaching in PE can be improved through additional training opportunities [17]. When PE teachers (especially generalists) do not receive experiences and content-training, they demonstrate lower levels of confidence to teach and plan PE [55]. At the preservice level, courses should be structured to ensure training and experiences related to PE; these will help preservice teachers develop familiarity and confidence with the PE curriculum [18]. Lower levels of confidence to teach PE among generalists has been cited as a concern on the part of school leadership [16]. The findings that specialists possessed more confidence to deliver lessons as a result of training and subject knowledge supports previous literature (e.g., [16,46]), further highlighting the need for additional training for teaching in PE.

It is important to acknowledge the limitations of this work. The scope of the present study did not account for FMS progress through specific teacher style interventions in PE, future research should focus on the practical relationship between the two. The intention was to collect 50 participants from each of the teaching groups for the quantitative portion; however, only 10 survey respondents identified as specialists. This may be due to the generalist model that has been widely adopted within Ontario, which may have limited the number of potential specialists that could be recruited. It is also important to note that Little’s MCAR test found 22.5% of responses were missing/incomplete; this is rather high, which may indicate that the survey is too long in length. A final limitation is the moderate reliability scores found in the current data; this is different from the findings of Kulinna and Cothran [21] which found highly reliable results. Further psychometric testing should be done to ensure the reliability of the survey instrument.

## 5. Conclusions

Our findings indicate that the reproductive cluster of teaching styles was the most used among PE teachers, and that the practice style (style B) was perceived the most favourably by all instructors, with specialist teachers possessing the strongest views. Findings highlight a preference for a collaborative approach to teaching and learning, which depends on teacher confidence with subject material and their abilities to deliver lessons that effectively target the development of FMS. Together, examination of teachers’ self-reported use of the STS and preferences for implicit or explicit learning activities provide a holistic view of teaching FMS in primary school PE, and promote an understanding of the differences between teacher groups.

Further research for teaching in PE should focus on the relationship between teaching styles and FMS development, specifically within the PE program, and should continue to explore the different teaching approaches for PE possessed by different teacher groups (preservice, generalist, and specialist; e.g., [46]). Research should continue to distinguish between teacher groups and compare teaching styles among these groups to determine which teaching styles are used most commonly amongst the different PE teachers. A further area of interest would be to compare the actual teaching styles of these teacher groups and compare self-reported and actual use of the STS to promote FMS development. Finally, as mentioned in the Limitations section, further development to the STS questionnaire [21] should be made to ensure reliability remains consistent between uses.

## Figures and Tables

**Table 1:** Participant frequencies.

	Frequency	%
Age		
18–25	42	41.2
>25	60	58.8
Gender		
Male	32	31.4
Female	70	68.6
Setting		
Urban	46	45.1
Suburban	40	39.2
Rural	10	9.8
Not specified	6	
Teacher Type		
Preservice	38	37.3
Generalist	54	52.9
Specialist	10	9.8
*N* = 102		

**Table 2:** Multiple regression results for reproductive cluster (preservice, generalist, and specialist teachers).

Reproductive	*B*	95% CI for *B*		*SE B*	β	*R* ^2^	Δ*R*^2^
		*LL*	*UL*				
Model						0.118	0.109
Constant	3.253 ***	3.008	3.497	0.123			
Teacher Type	0.238 ***	0.105	0.371	0.067	0.344 ***		
Age					0.072		
Gender					0.034		
Setting					−0.064		

Note. Model = “Stepwise” method in SPSS Statistics; *B* = unstandardized regression coefficient; *CI* = confidence interval; *LL* = lower limit; *UL* = upper limit; *SE B* = standard error of the coefficient; β = standardized coefficient; *R*^2^ = coefficient of determination; Δ*R*^2^ = adjusted *R*^2^; * *p* < 0.05, ** *p* < 0.01, *** *p* < 0.001.

**Table 3:** Descriptive statistics for reproductive cluster (preservice, generalist, and specialist teachers).

Reproductive	Preservice		Generalist		Specialist	
	*M*	*SD*	*M*	*SD*	*M*	*SD*
Style A	3.38	0.634	3.66	0.685	3.84	545
Style B	4.10	0.466	4.25	0.453	4.39	0.582
Style C	3.71	0.397	3.75	0.633	4.08	0.422
Style D	2.77	0.838	3.02	0.822	3.37	0.887
Style E	3.68	0.651	3.79	0.671	4.26	0.615

Note. *M* = mean, *SD* = standard deviation.

**Table 4:** Multiple regression results for reproductive (preservice and in-service teachers).

Productive	*B*	95% CI for *B*		*SE B*	β	*R* ^2^	Δ*R^2^*
		*LL*	*UL*				
Model						0.067	0.058
Constant	3.285 ***	2.984	3.586	0.152			
Teacher Type	0.232 **	0.055	0.408	0.089	0.260 **		
Age					0.079		
Gender					0.020		
Setting					−0.079		

Note. Model = “Stepwise” method in SPSS Statistics; B = unstandardized regression coefficient; CI = confidence interval; LL = lower limit; UL = upper limit; SE B = standard error of the coefficient; β = standardized coefficient; R^2^ = coefficient of determination; ΔR^2^ = adjusted R^2^; * *p* < 0.05, ** *p* < 0.01, *** *p* < 0.001.

**Table 5:** Descriptive statistics for reproductive cluster (preservice and in-service teachers).

Reproductive	Preservice		In-Service	
	*M*	*SD*	*M*	*SD*
Model				
Style A	3.38	0.634	3.69	0.665
Style B	4.10	0.466	4.27	0.473
Style D	2.77	0.838	3.07	0.835
Style E	3.68	0.651	3.86	0.680

Note. *M* = mean, *SD* = standard deviation.

## Data Availability

Quantitative data are available upon reasonable request from the corresponding author; however, ethics clearance limits the distribution of qualitative interview data beyond the representative quotes that appear in this paper.
